# Forty-Nanometer Plasmonic Lithography Resolution with Two-Stage Bowtie Lens

**DOI:** 10.3390/mi14112037

**Published:** 2023-10-31

**Authors:** Yan Meng, Ruiguang Peng, Jie Cheng, Yonggang Meng, Qian Zhao

**Affiliations:** 1 School of Mechanical and Electronic Engineering, China University of Mining and Technology, Beijing 100083, China; mengyan416@163.com; 2 Department of Mechanical Engineering, State Key Laboratory of Tribology, Tsinghua University, Beijing 100084, China; mengyg@tsinghua.edu.cn; 3 Institute of New Materials and Advanced Manufacturing, Beijing Academy of Science and Technology, Beijing 100084, China; pengruiguang@bjast.ac.cn

**Keywords:** plasmonic lens, maskless nanolithography, bowtie aperture

## Abstract

Optical imaging and photolithography hold the promise of extensive applications in the branch of nano-electronics, metrology, and the intricate domain of single-molecule biology. Nonetheless, the phenomenon of light diffraction imposes a foundational constraint upon optical resolution, thus presenting a significant barrier to the downscaling aspirations of nanoscale fabrication. The strategic utilization of surface plasmons has emerged as an avenue to overcome this diffraction-limit problem, leveraging their inherent wavelengths. In this study, we designed a pioneering and two-staged resolution, by adeptly compressing optical energy at profound sub-wavelength dimensions, achieved through the combination of propagating surface plasmons (PSPs) and localized surface plasmons (LSPs). By synergistically combining this plasmonic lens with parallel patterning technology, this economic framework not only improves the throughput capabilities of prevalent photolithography but also serves as an innovative pathway towards the next generation of semiconductor fabrication.

## 1. Introduction

Fabricating ultra-fine nanoscale patterns with high throughput is essential for a wider range of applications in high-speed computing, data storage, nanofabrication, sensing, light-matter interaction enhancement, surface-enhanced Raman scattering, and nanophotonics [1]. Currently, high-end semiconductor components, are the pivotal nuclei of intelligent manufacturing, and large-scale integrated circuits have permeated into every aspect of the national economic proposal. The evolutionary trajectory of chip manufacturing techniques has perpetually adhered to the tenets of Moore’s Law. At present, conventional photolithography approaches have drawn nearer to the diffraction limits intrinsic to light [2,3]. It is well understood that the conventional interference-lithography’s resolving power is restricted to approximately λ/2, wherein λ signifies the wavelength of incident light. To further improve the photolithographic resolution, it is necessary to further shrink the wavelength of incident light or to utilize the complex technology such as multiphoton absorption and multiple exposures. Thus, a reasonable and inevitable solution emerges to adopt deep ultraviolet (DUV), extreme ultraviolet (EUV), and even soft X-ray sources for exposure [4]. However, the technical difficulties and high costs associated with these sources have constrained the widespread utilization of nano-lithographic technology [5,6]. However, these technologies need masks to accomplish nanofabrication. Because of the ever-increasing complexity and cost of the mask-based lithography, unmasked schemes are emerging as viable approaches, and eliminating the need for masks reduces cost and design cycle [7]. However, the low throughput of most unmasked methods due to the serial and slow scanning nature remains the bottleneck. Therefore, significant improvements in maskless lithography are essential to satisfy the demands of the semiconductor industry for mass production. Working in the optical near-field is another way to overcome the resolution limitations of conventional lithography. However, it still faces some vital obstacles, such as energy throughput and working distance control required for high-volume manufacturing.

Surface Plasmon (SP) is a type of evanescent wave, an oscillation collectively excited by free electrons at the interface between dielectric and metal. Delimited by the dielectric-metal interface, it boasts a lateral wave vector greater than that of light waves [8,9]. The intrinsic cause behind the Abbe diffraction limit lies in the exponential decay exhibited by evanescent waves that are the bearers of sub-wavelength information, such as fine features, within the medium, and thereby resulting in information loss and image imperfections [10]. Based on the localized field enhancement and sub-wavelength confinement attributes intrinsic to surface plasmonic lenses, sub-diffraction resolution imaging capabilities have been manifested in lots of nano-lithographic techniques, including plasmonic lithography, lens-parallel lithography, and near-field lithography [10,11]. Plasmonic lithography, characterized by its maskless nature, cost-effectiveness, and potential to attain ultra-high resolution, has received extensive research attention [1,6,12]. Theoretical and experimental outcomes uniformly underscore that the resolution of plasmonic lithography can descend near 50 nm. However, owing to the exponential attenuation characteristic of surface plasmon lenses in the direction of patterned depth, nearly all plasmonic lithography techniques yield relatively shallow pattern depths. Furthermore, it has been demonstrated in the literature that the addition of a metal layer under the photoresist can increase the etching depth [13,14,15].

To attain lithographic patterns characterized by high fidelity and aspect ratios, we ingeniously incorporate the bowtie junction structure alongside an annular grating [16,17]. This strategic combination of Localized Surface Plasmons (LSPs) and Propagating Surface Plasmons (PSPs) ultimately created a groundbreaking plasmonic lens. Thus, we achieved an awe-inspiring resolution of 40 nm through lithography, facilitated by a 355 nm laser source, thereby transcending the boundaries of optical diffraction limits. This seminal advancement will have a profound proliferation of applications in nanofabrication.

## 2. Plasmonic Lens Design

Generally incident light cannot excite SP on the metal surface directly due to the momentum mismatch between the light waves and the SP. However, by matching these momentums, typically using a rough surface, grating coupler, or attenuated total reflection (ATR) coupler, incident light can be converted into SP.

In this context, the utilization of a ring grating to incite SP is merited, owing to its structural simplicity and simple preparatory nature. The annular grating must adhere to the below equation to harmonize with the wave vector of SP.

(1) $$k_{sp}=k_{0}\sin\theta_{i}+vg$$

where *g* is defined as 2*π*/*a*, signifying the magnitude of the reciprocal lattice vector for a periodicity of *a*; *v* = (1, 2, 3, …); *k*_0_ represents the wave vector of electromagnetic waves in free space, while *θ_i_* stands for the angle of incidence.

The interaction of light and SP is described by the SP dispersion relation (the frequency-dependent SP wavevector, *k_SP_*) [18]:

(2) $$k_{sp}=k_{0}\sqrt{\frac{\varepsilon_{d}\varepsilon_{m}}{\varepsilon_{d}+\varepsilon_{m}}}$$

where *k*_0_ is the wavevector of light in a vacuum; *ε_m_* and *ε_d_* are dielectric constants of the metal and the surrounding dielectric material, respectively. Amalgamating expressions (1) and (2), one can calculate the requisite grating constant for the annular grating. Through the SP dispersion curve, it is apparent that the SP wavelength exhibits a marked reduction when compared with that of incident light. Consequently, this phenomenon will engender finer resolution in plasmonic lithography.

In general discourse, because of the larger propagation wave vector of SP in comparison to light waves, the excitation of SP requires the involvement of light waves. This process is facilitated by the strategic design of an annular grating, effectively manipulating free-space light for the controlled excitation of SP. The resultant propagation of SP takes place at the interface of metal and dielectric, guided by the configuration of the annular grating, thus achieving a focused propagation of surface plasmons.

A 355 nm ultraviolet laser is chosen as the light source. At ultraviolet wavelengths aluminum is a good candidate for metallic material due to its relatively low losses. Nonetheless, the mechanical and adhesion properties of aluminum are poor, which could cause the mechanical damage of lenses during lithography. Considering the manufacturing cost and reproducibility of the flying head, just like the mask in the photolithography technology, it would be better to choose the chromium for its mechanical and adhesion properties. With its commendable processing characteristics, the chromium layer provides a preferable alternative for exciting SP.

In this context, two commonplace lens structures are devised and compared: the bowtie-aperture lens and the circular-aperture lens. They are coupled with annular symmetrical gratings and semicircular asymmetrical gratings, respectively, and simulations are performed to calculate the near-field distributions.

**Figure 1:**
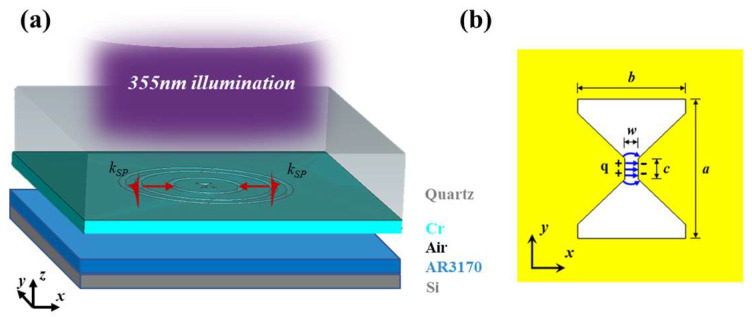
(**a**) Schematic picture of plasmonic lithography; (**b**) the structure and electric-field distribution of bowtie aperture.

The pivotal two-stage lens (TSL) design to achieve the high resolution consists of nano-apertures, a set of ring couplers (two inner rings) and a ring reflector (the outer ring), fabricated on a metallic thin film. As schematically illustrated in Figure 1a, the PSPs are excited and propagate towards the center of TSL using a circular shape grating.

When ultraviolet light impinges upon the annular grating on the metallic film, the scattering induced by the edges of the grating grooves converts a portion of the incident light into SP, with the direction of energy transmission of SP being perpendicular to the grooves. Thus, this annular groove has the capacity to conduct and converge SP energy to the focal point at the center of the ring, requiring only that the period between adjacent rings be equal to the wavelength of the SP. This alignment ensures that the SP generated by different rings undergoes constructive interference at the focal point of the lens.

At the focal point of TSL, these PSPs are transformed into deep subwavelength LSPs through the nano-apertures (circular or bowtie-shaped aperture). In the realm of circular-aperture lenses, oscillating electrons on the surface of metal nanoparticles tend to concentrate at the edges. This phenomenon begets a scenario wherein the field intensity at the periphery of the circular lens surpasses that at its center, thereby engendering a trough of field intensity at the central point. The bowtie aperture, a commonly employed metallic nanostructure, falls within the realm of localized surface plasmon resonances. The configuration of the bowtie aperture, as depicted in Figure 1b, exhibits a remarkable ability to confine the electric field within the gap as substantially smaller than the incident wavelength [17,19,20,21]. Positive and negative charges converge upon the metallic ridges on opposing sides, yielding an arrangement of an electric dipole moment, thus engendering the foundational mode of LSPs. In this depiction, the polarity signifies the distribution of charge, while the blue arrows denote the direction of the electric field. The near-field lithographic resolution of bowtie aperture is decided using its gap size [22]. Guided by the annular grating, PSPs engender a notably amplified energy transmission magnitude, culminating around the core of the bowtie-aperture structure. This deliberate architectural arrangement generates heightened optical energy, surpassing the intensity of conventional LSPs, and thereby holds promise for achieving superior pattern refinement.

In pursuit of further compressing the spot size at the focal point, an asymmetrical annular grating is conceived, adjusting the outward radial displacement of the right-sided annular grating by *λ_SP_*/2. In this movement, the optical path traversed by the surface plasmons engendered on the right side, transmitting to the focus, bears a phase difference of half a wavelength compared to the left-sided surface plasmons. This orchestrated phase discrepancy results in a phase-cancellation interference at the focal point.

**Figure 2:**
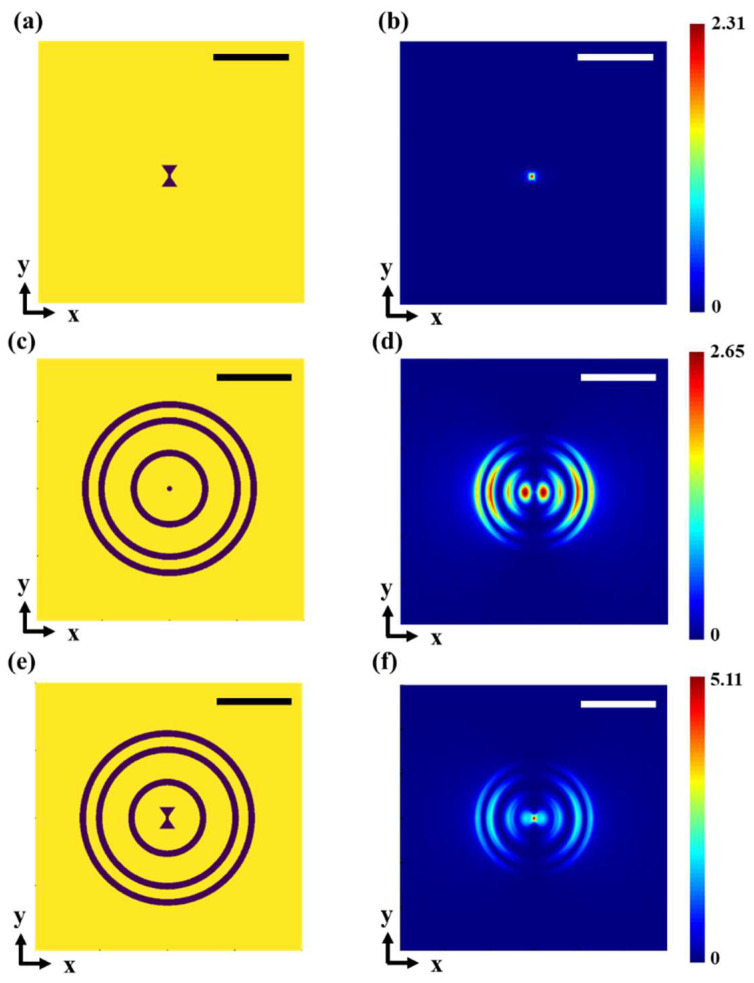
Designs of symmetric plasmonic lens (PL) structures and simulation results. Three cases of PL designs are shown here for comparison. (**a**) Single bowtie aperture; the optimal parameters of bowtie aperture are *a* = 160 nm, *b* = 120 nm, *c* = 20 nm, *w* = 10 nm. (**b**) FDTD simulation results of (**a**); (**c**) 40 nm diameter circular aperture with ring grating and an additional outer-ring reflector; (**d**) FDTD simulation results of (**c**); (**e**) bowtie-shaped aperture with ring grating and an additional outer ring reflector; (**f**) FDTD simulation results of (**e**); and both scale bars are 500 nm.

Through electromagnetic simulation, we compared two plasmonic-structure designs working at the excitation wavelength of 355 nm with their corresponding light intensity normalized by the incident light intensity. Figure 2c shows an example of PSP-based PL with a 40 nm diameter circular aperture at its center. By guiding the PSP energy towards the center, it is capable of producing orders of a magnitude of higher energy intensity through the same-size hole. However, it can be seen that the net intensity through a PSP-based PL decreases rapidly when the center hole size further reduces into the deep sub-wavelength region. Figure 2e shows another TSL design where the center hole is replaced by a bowtie-shaped aperture in order to further enhance the confinement and energy intensity at the focal point.

All of the optimal parameters were investigated with the finite-difference time-domain (FDTD) software (CST studio suite 2020), including the grating radius, grating width, and metal-film thickness. The refractive index of the quartz wafer and chromium film were set as 1.48 and 1.39 + 3.24*i*, respectively. For the etched part, the refractive index was set to unity. The boundary condition of the quartz was chosen as a perfect match layer (PML), which is expressed as ***n*** × **E** = **0**, meaning that the electric field is perpendicular to the surface of the quartz and electromagnetic waves are completely absorbed; therefore, the tangential component of the electric field is equal to zero. The boundary condition of the others was set as the PML, ***n*** × (**∇** × **E**) − *jk***n** × (**E** × ***n***) = **0**, implying that the electromagnetic waves are completely absorbed. The metal-film surface was illuminated from the quartz with a linearly polarized beam with a wavelength of 355 nm. The whole simulation region of the model, 3 μm × 3 μm × 0.3 μm, was discretized into 59,172,000 elements, with a minimum element unit of 0.1 nm. The reference plane was set 15 nm below the air–metal interface. The single-variable factor method was used during all of the simulations.

## 3. Simulation Results

**Figure 3:**
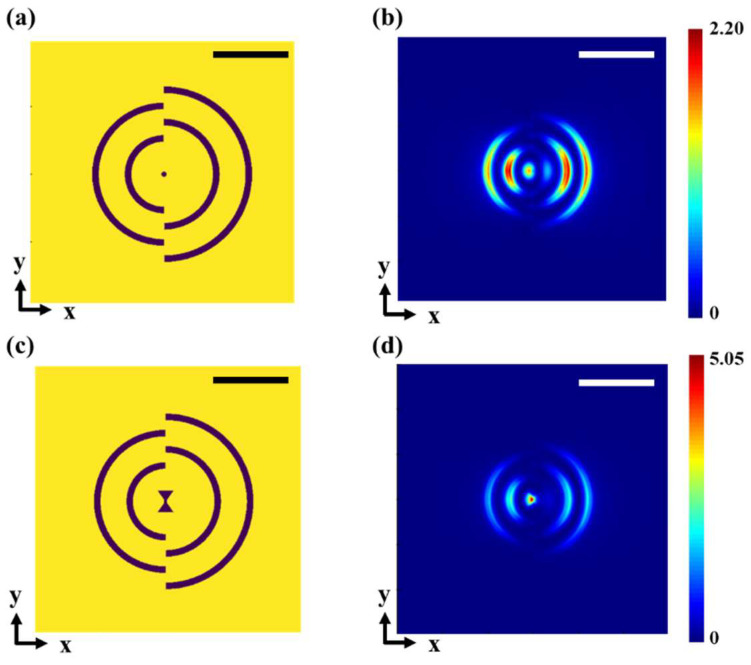
Designs of asymmetric plasmonic lens (PL) structures and simulation results. Two cases of PL designs are shown here for comparison. (**a**) Forty nm diameter circular aperture with semicircular asymmetrical gratings; (**b**) simulated 2D electromagnetic-field distribution of (**a**); (**c**) bowtie-shaped aperture with semicircular asymmetrical gratings, and the optimal parameters of bowtie aperture are *a* = 160 nm, *b* = 120 nm, *c* = 20 nm, *w* = 10 nm; (**d**) simulated 2D electromagnetic-field distribution of (**c**); and both scale bars are 500 nm.

The simulated results for plasmonic lens (PL) configurations unveil the electric-field distribution within the plane located 15 nm below the metallic thin film. The structures and simulation results of two apertures with ring grating and an additional outer-ring reflector are shown in Figure 2. Primarily, simulations were conducted concerning the solitary bowtie-aperture lens, as depicted in Figure 2b. The discernible outcome reveals a marked augmentation in the focal field strength of the bowtie lens, attributed to the presence of the annular grating. In the case of the bowtie configuration, the optical spot size is 52 nm, accompanied by a peak electric-field intensity reaching 5.11 times of the incident-light intensity. Meanwhile, the circular configuration exhibits an optical spot size of 105 nm, with the highest point of electric-field intensity measuring 2.65 times the incident-light intensity. The simulation results of anti-symmetric gratings 15 nm below the metallic thin film are shown in Figure 3. In the case of the bowtie configuration, the optical spot size is 64 nm, accompanied by a peak electric-field intensity reaching 5.05 times of the incident-light intensity. Meanwhile, the circular configuration exhibits an optical spot size of 95 nm, with the peak electric-field intensity measuring 2.20 times of the incident-light intensity.

**Figure 4:**
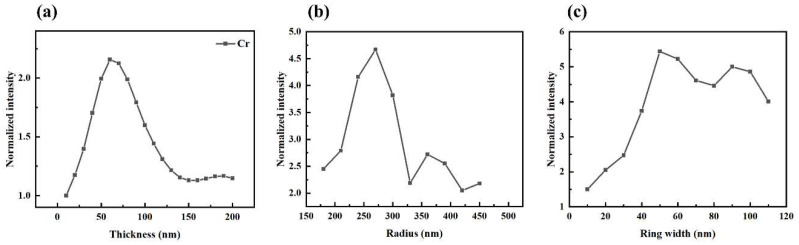
Simulation optimization of electric-field intensity of bowtie aperture for (**a**) different Cr metal-film thicknesses, (**b**) different grating radius, and (**c**) different grating widths.

It is noteworthy that the bowtie configuration surpasses the circular counterpart in terms of both focal-spot dimensions and central electric-field intensity. The parameters of the bowtie aperture, including the metal-film thickness, radius of the ring grating, and width of the grating slits, have all been meticulously optimized. The outcomes of the parameter scan are illustrated in Figure 4.

The simulation results for an equivalent lens with an aluminum metal layer are depicted in Figures S1–S5, wherein both the intensity of the focal spot and the full width at half maximum (FWHM) exhibit superior attributes in comparison to metallic chromium. However, considering the mechanical damage and reproducibility of the flying head, the selection of metallic chromium is favored as the material due to better mechanical and adhesion properties.

**Figure 5:**
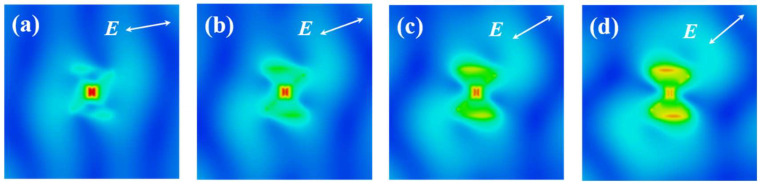
The electromagnetic-field distribution of the lens with various angularly polarized light incidences; (**a**) 10°, (**b**) 20°, (**c**) 30°, and (**d**) 40°.

In the course of simulation, a rigorous adherence was maintained to ensure the x-direction polarization of incident light, precisely perpendicular to the bowtie aperture placed at the center of the bowtie structure. However, during the experimental section, the presence of components parallel to the ridge aperture’s central slit emerged within the incident light due to machining errors and equipment misalignments. To study the influence of polarization direction on the focal-spot energy of the lens, the electromagnetic-field distribution of the lens was computed for various angularly polarized light incidences, as depicted in Figure 5. The white arrow in the upper right corner designates the polarization direction, with polarization angles of 10°, 20°, 30°, and 40°, respectively. As the polarization angle increases, the field intensity at the center of the bowtie aperture gradually diminishes. Furthermore, when the polarization angle surpasses 20°, a conspicuous enhancement of the localized electric field manifests at the upper and lower boundaries of the bowtie aperture, engendering a dispersal of focal field energy and a concomitant deterioration in the focusing effect.

**Figure 6:**
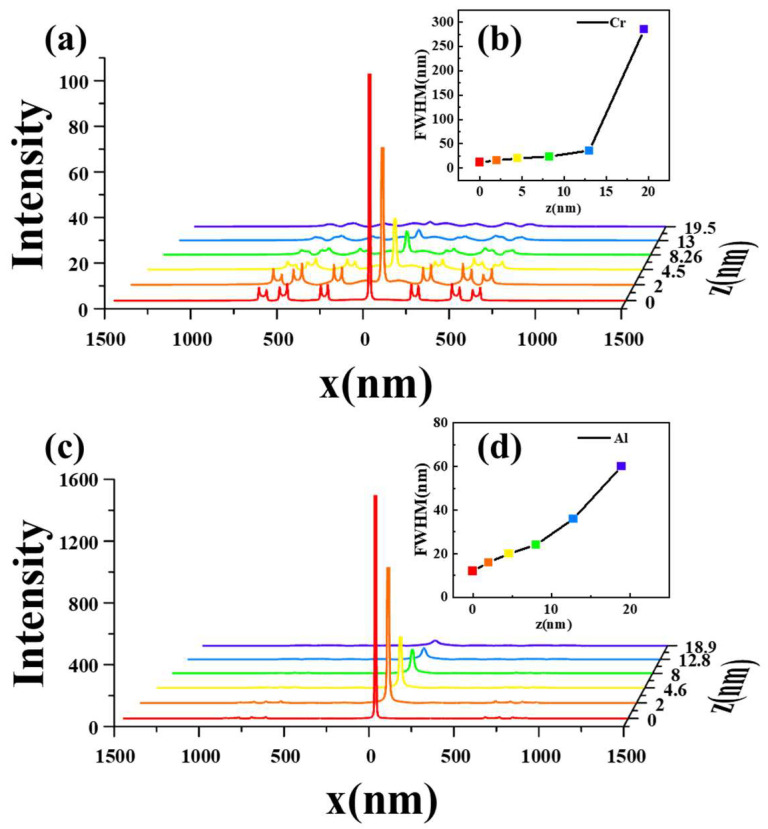
(**a**) Intensity of SP at different z underneath the Cr metal film and (**b**) the full width at half maximum of the focal spot; (**c**) intensity of SP at different z underneath the Al metal film; and (**d**) the full width at half maximum of the focal spot.

In addition to the field distribution located 15 nm beneath the metallic layer, the variations in the size of the focused light spot and its intensity with different distances at z-direction from the center of the bowtie-aperture slit were shown in Figure 6. Given that the energy of surface plasmons experiences exponential attenuation with distance, this pattern can provide guidance for controlling the distance between the lens and the photoresist during the experimental process, thereby yielding enhanced plasmonic lithographic outcomes.

The simulation results elucidate the supremacy of the bowtie aperture over the circular-aperture lens design. The conjunction of the ring grating with the bowtie aperture imparts a superior focal intensity and a narrower full width at half maximum.

## 4. Methods

**Figure 7:**
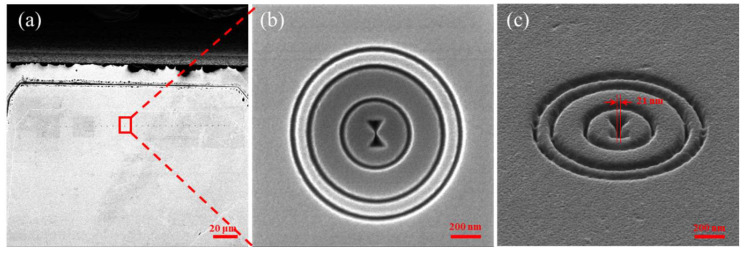
SEM image of FIB results of bowtie aperture: (**a**) arrangement of PL; (**b**) the structure of single PL; and (**c**) side view of PL structure.

Upon the surface of a quartz substrate, a 60 nm thick chromium metal film was deposited using electron-beam evaporation (anelva L-400EK, Kawasaki, Japan). The designed plasmonic lenses (PL) were then fabricated using Zeiss triple-beam focused-ion-beam (FIB, zeiss orion nanofab, Jena, Germany) microscope. Considering both etching efficiency and structural accuracy, the circle grating was processed using a high-energy Ga-FIB with an accuracy of 20 nm, while the 20 nm bowtie structure, demanding higher precision, was processed using He-FIB. The smallest gap size that can be realized in this work is 21 nm, as shown in Figure 7.

**Figure 8:**
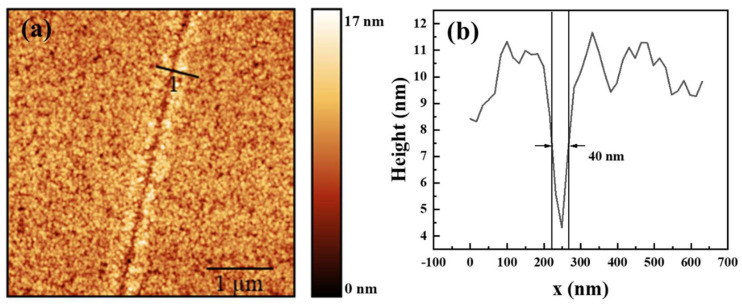
AFM image of plasmonic lithography of single bowtie-aperture lens: (**a**) 2D picture; and (**b**) the full width at half maximum of lithographic resolution.

For the lithography process, a 60 nm thick photoresist AR3170 was uniformly spun on the surface of the silicon substrate, maintaining roughness within 1 nm. The metal block with the PL pattern was affixed to a cantilever, and 355 nm ultraviolet light was illuminated, and focused down to a few micrometer spots on top of the PL while rotating the Si disk to achieve exposure. After the pattern exposure followed by 2.38% tetramethylammonium hydroxide (THAM) development, the resulting features were then examined using an atomic force microscope (AFM, bruker icon dimension, Germany) and shown in the Figure 8.

## 5. Conclusions

In summary, the combination of the ring grating with the bowtie aperture imparts a superior focal intensity and a narrower FWHM. Based on the simulation results, we have demonstrated a high-speed plasmonic nanolithography with 40 nm resolution. This is achieved by employing two-stage plasmon focusing through relatively low-loss propagating surface plasmons focusing, and later converting to, localized plasmons. 

This new scheme enables a low-cost, high-throughput unmasked nano-scale fabrication with a few orders of magnitude higher throughput than conventional unmasked approaches. It may allow continuous scaling to a smaller node size beyond 40 nm by utilizing shorter SPs wavelength and decreasing the air gap between SP lenses and photoresist. The present method can be easily realized with a 355 nm pulsed laser, which is more advantageous than other fabrication methods. It opens up a promising route towards next-generation lithography for semiconductor manufacturing.

## Data Availability

The data presented in this study are available on request from the corresponding author.

## Supplementary Materials

The following supporting information can be downloaded at: https://www.mdpi.com/article/10.3390/mi14112037/s1, Figure S1: Simulation results of electric field intensity of bowtie aperture at 15 nm for different Al metal film thicknesses; Figure S2: Simulation results of electric field intensity of bowtie aperture at 15 nm for different grating radius; Figure S3: Simulation results of electric field intensity of bowtie aperture at 15 nm for different grating widths; Figure S4: Designs of plasmonic lens (PL) structures and simulation results. Two cases of PL designs are shown here for comparison, including: (a) 40-nm diameter circular aperture with ring grating and an additional outer ring reflector; (b) FDTD simulation results of (a); (c) bowtie-shaped aperture with ring grating and an additional outer ring reflector, the optimal parameters of bowtie aperture are *a* = 160 nm, *b* = 120 nm, *c* = 20 nm, *w* = 20 nm; (d) FDTD simulation results of (c); both scale bars are 500 nm.; Figure S5: Designs of plasmonic lens (PL) structures and simulation results. Two cases of PL designs are shown here for comparison, including: (a) 40-nm diameter circular aperture with semicircular asymmetrical gratings; (b) simulated 2D electromagnetic field distribution of (a); (c) bowtie-shaped aperture with semicircular asymmetrical gratings, the optimal parameter of bowtie aperture are *a* = 160 nm, *b* = 120 nm, *c* = 20 nm, *w* = 20 nm; (d) simulated 2D electromagnetic field distribution of (c); both scale bars are 500 nm.
