# Supplementary material for: Forty-Nanometer Plasmonic Lithography Resolution with Two-Stage Bowtie Lens
Source: Micromachines (Basel). 2023 Oct 31;14(11):2037. doi: 10.3390/mi14112037 (PMC10673134; doi:10.3390/mi14112037)
Supplement: Supplementary file 1 [file micromachines-14-02037-s001.zip › micromachines-2610815-supplementary.pdf]

Supplementary Materials

Forty-Nanometer Plasmonic Lithography Resolution with Two-Stage Bowtie Lens

Yan Meng [1,2], Ruiguang Peng [3], Jie Cheng [1,*], Yonggang Meng [2] and Qian Zhao [2,*]

1 School of Mechanical and Electronic Engineering, China University of Mining and Technology, Beijing 100083, China; mengyan416@163.com

2 Department of Mechanical Engineering, State Key Laboratory of Tribology, Tsinghua University, Beijing 100084, China; mengyg@tsinghua.edu.cn

3 Institute of New Materials and Advanced Manufacturing, Beijing Academy of Science and Technology, Beijing 100084, China; pengruiguang@bjast.ac.cn

* Correspondence: jiecheng@cumtb.edu.cn (J.C.); zhaoqian@mail.tsinghua.edu.cn (Q.Z.)

All of the optimal parameters were investigated by using FDTD method with the software CST studio suite 2020, including the grating radius, grating width, and metal film thickness. The refractive index of aluminum film was set as *0.385+4.30i*. The simulation results were shown in Figure S1-S3.

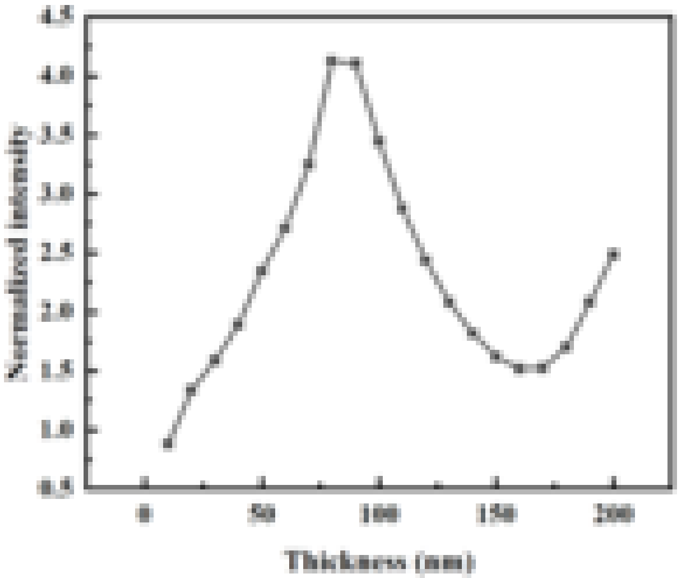

Figure S1. Simulation results of electric field intensity of bowtie aperture at 15 nm for different Al metal film thicknesses.

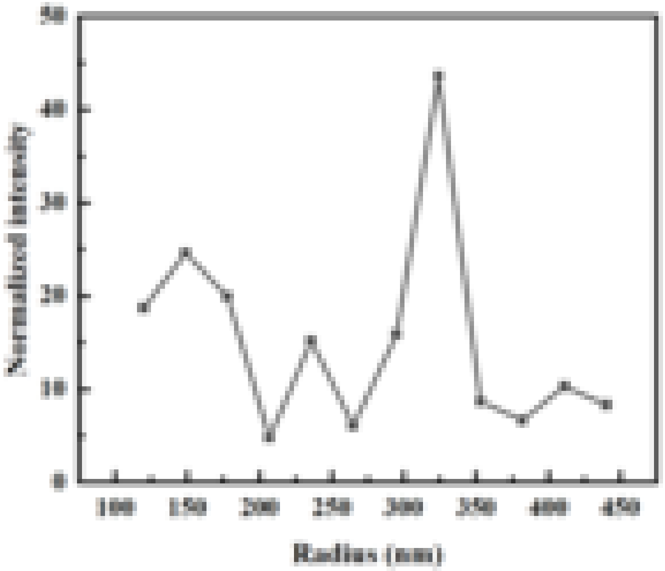

Figure S2. Simulation results of electric field intensity of bowtie aperture at 15 nm for different grating radius.

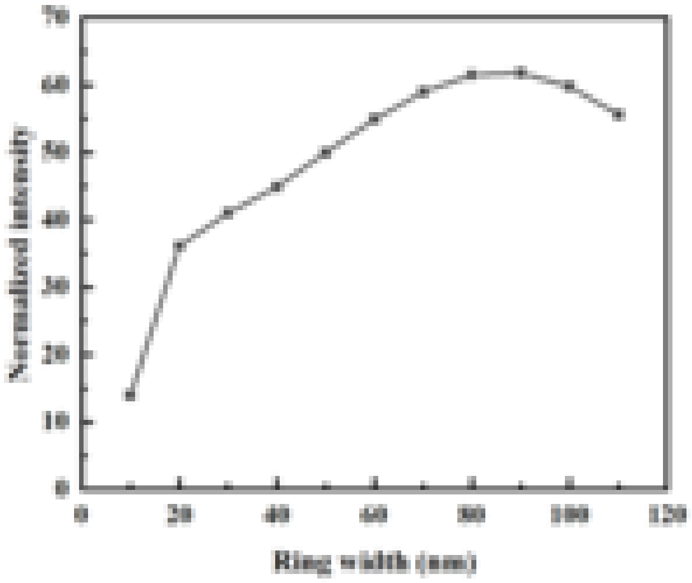

Figure S3. Simulation results of electric field intensity of bowtie aperture at 15 nm for different grating widths.

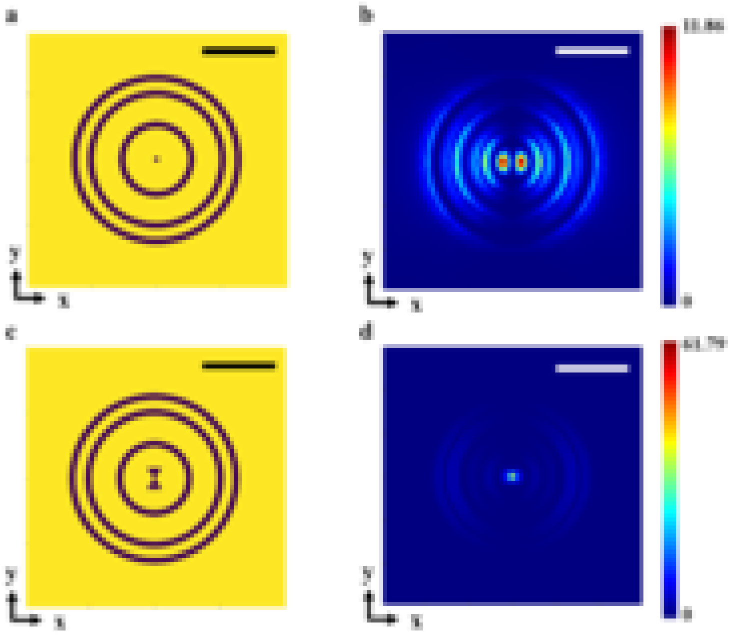

Figure S4. Designs of plasmonic lens (PL) structures and simulation results. Two cases of PL designs are shown here for comparison, including: (a) 40-nm diameter circular aperture with ring grating and an additional outer ring reflector; (b) FDTD simulation results of (a); (c) bowtie-shaped aperture with ring grating and an additional outer ring reflector, the optimal parameters of bowtie aperture are a = 160 nm, b = 120 nm, c = 20 nm, w = 20 nm; (d) FDTD simulation results of (c); both scale bars are 500 nm.

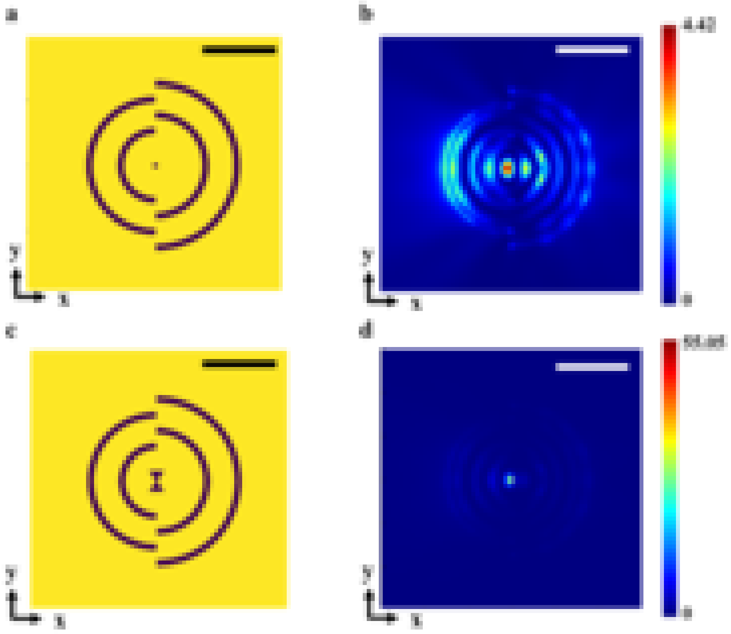

Figure S5. Designs of plasmonic lens (PL) structures and simulation results. Two cases of PL designs are shown here for comparison, including: (a) 40-nm diameter circular aperture with semicircular asymmetrical gratings; (b) simulated 2D electromagnetic field distribution of (a); (c) bowtie-shaped aperture with semicircular asymmetrical gratings, the optimal parameters of bowtie aperture are a = 160 nm, b =

120 nm, c = 20 nm, w = 20 nm; (d) simulated 2D electromagnetic field distribution of (c); both scale bars are 500 nm.
